# Using HRS-ONET Linked Data to Study Subjective and Objective Mismatch Between Work Demands and Capacity at Older Ages

**DOI:** 10.1093/geroni/igab046.888

**Published:** 2021-12-17

**Authors:** Brooke Helppie-McFall, Amanda Sonnega, Gwen Fisher

**Affiliations:** 1 University of Michigan, Ann Arbor, Michigan, United States; 2 Colorado State University, Fort Collins, Colorado, United States

## Abstract

Mismatch between demands of work and workers’ ability to meet those demands may play an important role in retirement decisions. This presentation extends earlier work using Health and Retirement Study data linked to O*NET to develop measures of discrepancy between individual’s own reports of physical and mental abilities and 1) their perceptions of the physical and mental demands of their jobs and 2) O*NET ratings of the physical and mental demands of their jobs. In particular, we utilize newly available linked information using 2010 Census codes and 2019 O*NET ratings that reflect more current jobs. We then examine the impact of each type of mismatch (subjective and objective) on retirement timing. Overall, we find a stronger connection between subjective mismatch relative to objective mismatch. We discuss implications of this finding in terms of the value of the O*NET linkage and potential interventions aimed at extending working lives for positive aging.

